# Dual role of autoantibodies to heat shock proteins in autoimmune diseases

**DOI:** 10.3389/fimmu.2024.1421528

**Published:** 2024-06-06

**Authors:** Stefan Tukaj

**Affiliations:** Department of Molecular Biology, Faculty of Biology, University of Gdańsk, Gdańsk, Poland

**Keywords:** autoantibodies, heat shock proteins, Hsp, autoimmunity, immunoregulation

## Abstract

Autoimmune diseases are characterized by the recognition of self-antigens (autoantigens) by immune system cells. Loss of immunological tolerance may lead to the generation of autoantibodies and, consequently, tissue damage. It has already been proven that highly immunogenic bacterial and autologous extracellular heat shock proteins (eHsps) interact with immune cells of the innate and adaptive arms of the immune system. The latter interactions may stimulate a humoral (auto)immune response and lead to the generation of anti-Hsps (auto)antibodies. Although circulating levels of anti-Hsps autoantibodies are often elevated in patients suffering from multiple inflammatory and autoimmune diseases, their role in the development of pathological conditions is not fully established. This mini-review presents the dual role of anti-Hsps autoantibodies - protective or pathogenic - in the context of the development of selected autoimmune diseases.

## 1 Introduction

Autoimmune diseases (ADs) are characterized by the recognition of self-antigens (autoantigens) by immune system cells, which consequently leads to chronic inflammation and tissue damage. Although significant progress has been made in discovering the key factors involved in the pathophysiology of various autoimmune diseases, their therapy remains (in many cases) a challenge and often involves traditional immunosuppressive treatments, using corticosteroids or cytostatic drugs, or more advanced biological therapies targeting (i) specific cells of the immune system (e.g. anti-CD20 therapy), (ii) signaling molecules (e.g., JAK-STAT inhibitors), or (iii) cytokines (e.g., anti-TNF therapy) directly or indirectly involved in the development and maintenance of chronic inflammation. These therapies focus on suppressing inflammation; nevertheless, immunological tolerance and stable immunological balance are not achieved. Additionally, most currently available therapies cause numerous side effects. Therefore, there are constant efforts to better understand the pathophysiology of autoimmune diseases to develop more effective and safer therapies for these diseases (1). B cells appear to play a key role in the development of autoimmunity because they act as antigen-presenting cells to autoreactive T lymphocytes and are responsible for the production of pathogenic autoantibodies. On the other hand, numerous studies have shown that healthy individuals have detectable levels of circulating autoantibodies, which may suggest that autoantibody positivity is not necessarily associated with pathology. The question arises whether the mechanisms leading to the formation of naturally occurring auto-polyreactive (auto)antibodies (NAbs) are the same as those leading to the secretion of pathogenic autoantibodies? One theory is that chronic activation of the immune system may lead to the expansion of NAbs, which in turn may contribute to the development of autoimmune diseases in genetically predisposed individuals (2). This concept is in contradiction with the widely described protective role of NAbs (3, 4), but - as history shows - it cannot be completely ruled out.

The dual role of autoantibodies, i.e. protective or pathogenic, may involve autoantibodies directed against a highly conserved group of stress proteins, historically called “heat shock proteins” (Hsps), whose expression in cells can increase in response to various stress stimuli, including heat shock, oxidative stress, infection, or inflammation. In principle, intracellular Hsps may be released into the extracellular space by passive release from injured or necrotic cells, active secretion (e.g., by extracellular vesicles), or may be presented to T lymphocytes by antigen-presenting cells via major histocompatibility complex (MHC) molecules. Because autologous extracellular Hsps (eHsps) can activate both the innate and adaptive immune response, their presence in the extracellular space is often associated with the development of autoimmunity. However, the role of eHsps in these diseases is not clear because they have both pro- and anti-inflammatory effects. Determining the importance of eHsps in immune reactions is additionally complicated because higher titers of autoantibodies against Hsps are often reported in patients suffering from various inflammatory diseases (5–8). However, it is still unknown whether higher levels of antibodies to self-Hsps act as “friends” or “foes” in autoimmune diseases, and it appears to depend on several variables.

## 2 The role of heat shock proteins in cell biology

The cellular response to thermal stress (the heat shock response) was first described in Drosophila melanogaster by Italian researcher Ferruccio Ritossa in the early 1960s. This is undoubtedly a breakthrough discovery, but initially, it was not enthusiastically received by scientists. The manuscript describing this phenomenon was rejected by a very prestigious journal, where the editor indicated that the research had no biological significance. The results of the study were finally published in Experientia in 1962. Later, the heat shock response was correlated with the expression of Hsps, the presence of which was confirmed in all known organisms, from bacteria to humans. Hsps are among the most conserved proteins with a wide range of cellular functions described under both physiological and pathological conditions (9). Hsps have a chaperone or protease activity and are synthesized in various cellular compartments. Based on molecular weight, Hsps are classified into six major families, such as Hsp100, Hsp90, Hsp70, Hsp60, Hsp40, and small Hsp (sHsp) (10). In general, Hsps participate in the folding of newly synthesized polypeptides, refolding of denatured proteins, protein transport, proteolytic degradation, and stabilization of the structure of native proteins (11).

Protein quality control includes the regulation of protein synthesis, folding, and unfolding. This mechanism includes Hsps with chaperone or protease activity, as well as cellular clearance mechanisms including autophagy and the ubiquitin-proteasome system. When a protein escapes these mechanisms, misfolded forms accumulate in the cell, which can form aggregates that are toxic to the cell. Protein aggregates can also arise because of a failure of protein quality control machinery. Protein misfolding and aggregation are recognized as a hallmark of many human diseases, including neurodegenerative disorders. Although protein folding in a cell is a spontaneous process because its three-dimensional structure is determined by the sequence of amino acids in the polypeptide chain, many proteins require the help of molecular chaperones to fold properly and reach their native state. Molecular chaperones (such as Hsp70) assist in the folding and assembly of newly synthesized polypeptides by interacting with exposed hydrophobic residues of the polypeptides. The Hsp90 family, in turn, guards the basic structure of native proteins. The expression of both Hsp70 and Hsp90 can increase dramatically in a cell exposed to various stressors to maintain its proper functioning. Mechanically, stress stimuli that lead to the relaxation of the physiological structure of proteins and subsequent exposure of their hydrophobic regions lead to the induction of Hsps, which are involved in the refolding of denatured proteins. The molecular mechanism of Hsp induction and polypeptide folding has been comprehensively described in the cited review articles (12–14). Interestingly, numerous studies have shown that Hsps are overexpressed in inflamed tissues. However, it is not entirely clear whether Hsp induction in chronically inflamed tissues regulates the inflammatory process or is a factor contributing to the development of pathological conditions, including autoimmune diseases. It seems that the induction of Hsp in cells/tissues exposed to stress factors (e.g., ROS) should protect the cell against the adverse effects of these factors and activate repair mechanisms. Evidence of the regulatory activity of Hsp proteins is, for example, the immunosuppressive activity of intracellular Hsp70, which can inhibit the activity of NF-kB. However, intracellular Hsp90 has opposite properties in the context of the development of the inflammatory process because this chaperone promotes the activity of signaling factors (e.g. Jak-STAT) and transcription factors (e.g. NF-kB) involved in the inflammatory process, and pharmacological inhibition of Hsp90 activity leads to silencing of the inflammatory response, which has been proven in preclinical and clinical studies (5–8).

There are many proposed mechanisms explaining Hsps secretion (15), and if this is the case, the occurrence of eHsps cannot be merely an artifact. Increased or decreased eHsps levels in biological fluids (e.g., blood/serum) have been associated with many clinical conditions, which may either act as both pathological or protective factors or serve as biomarkers of the disease and indicators of its activity and progression. Initially, eHsps were suspected to have a strong pro-inflammatory effect and therefore their presence in the extracellular space was associated with the development of ADs. However, after many years it turned out that their inflammatory properties result mainly from the presence of endotoxins (including lipopolysaccharide, LPS), which have a strong affinity for Hsps. Removal of these contaminants from Hsps revealed in many (though not all) cases the opposite, i.e. immunosuppressive activity of eHsps. It is speculated that the mechanism of Hsps secretion plays a key role in the action of these proteins in the extracellular space in the context of the development of inflammatory diseases. For example, Hsps released from damaged cells can act as damage-associated molecular patterns (DAMPs) that activate the innate immune system by interacting with pattern recognition receptors (PRRs), while Hsps that form complexes with intracellular MHC I or MHC II molecules can lead to the activation of regulatory immune mechanisms. Autologous Hsps are believed to play an important role in the positive selection of T cells in the thymus, including the regulatory T cell fraction (5–8, 16–18).

Numerous studies demonstrating the immunosuppressive effects of Hsp-derived molecules are based on preclinical models of arthritis (18–20). For example, it was found that the highly conserved Hsp70 peptide (HSP70-B29) used for active immunization of animals could be considered as a potential target for the treatment of rheumatoid arthritis (RA) through the induction of Hsp70-specific regulatory T cells (Tregs) (21). Similarly, immunization of mice with Hsp70 resulted in a reduction in the clinical and histological severity of psoriasis in a mouse model, which was also associated with Treg expansion (22). Further evidence confirming the regulatory role of eHps in the functioning of the body is the widely reported role of eHsp90 in wound healing (23).

Regardless of the mode of Hsp secretion, highly immunogenic eHsps can lead to the activation of the humoral immune response and the production of specific autoantibodies. The question remains whether such antibodies have a protective effect, are part of the NAbs, or arise in response to tissue damage or other internal and external factors leading to the development of AD.

## 3 Two faces of autoantibodies

Immunoglobulins (Igs) are molecules produced by activated B lymphocytes and plasma cells in response to antigens. Igs mediate a variety of immune functions through interaction with antigens via the Ig Fab domain and cellular signaling triggered by the Ig Fc domain. Cell signaling via type I and type II Fcγ receptors is required for the control of inflammatory, but also anti-inflammatory, and immunomodulatory processes (24). Loss of immunological tolerance may lead to the development of autoantibodies and, consequently, tissue destruction (25). The occurrence of autoantibodies is a common feature of AD. They can be directed against various intra- and extracellular molecules, such as nucleic acids, lipids, glycoproteins, and proteins (26). These autoantibodies have become a useful tool in the diagnosis and monitoring of certain ADs, including systemic lupus erythematosus (SLE) or autoimmune bullous diseases (AIBD) (27, 28). Interestingly, anti-nuclear autoantibodies (ANA) or autoantibodies directed against structural skin proteins, commonly found in systemic autoimmune rheumatic diseases and AIBDs, respectively, are present in up to 10% of healthy people (29–32). It has been shown that these autoantibodies can persist in healthy people for several years before some people eventually develop symptoms of the disease (30, 33). There is increasing evidence that, like conventional Ig, IgG autoantibodies can have both pro-inflammatory and immunosuppressive effects. This is due to differences in the pattern of post-translational modifications (such as glycosylation/sialylation) of the IgG Fc regions and the IgG subclass (34, 35).

At a similar time, when the heat shock response was discovered, the presence of natural autoantibodies (NAbs) was proposed. Their occurrence was disregarded or denied by the immunological society due to their obvious contradiction with established immunological dogmas. NAbs are defined as germline-encoded immunoglobulins found in individuals without prior antigenic experience. They bind to exogenous (e.g. bacterial) or self-components and act as a first-line immune defense against infections, but also bind to and remove neo-autoantigens that are unmasked during tissue damage. According to many authorities in the field, however; such self-binding antibodies cannot be considered pathogenic autoantibodies in the classical sense for several reasons. First, disease-causing pathogenic autoantibodies undergo somatic mutations and, unlike NAbs, have high affinity and specificity. Also, NAbs have been shown to occur regardless of tissue damage or immunization. Finally, based on studies in mice and humans, NAbs are polyreactive and have low binding affinity to (self-)antigens. Unlike pathogenic autoantibodies, self-reactive natural IgM can reduce the incidence of autoimmune diseases (3, 4, 36).

## 4 Autoantibodies to self-Hsps in health and disease

Highly immunogenic bacterial and autologous eHsps have already been proven to interact with immune cells of the innate and adaptive arms of the immune system. The latter interactions may stimulate a humoral (auto)immune response and lead to the production of anti-Hsp (auto)antibodies. Although levels of anti-Hsp autoantibodies are elevated in patients suffering from numerous inflammatory and autoimmune diseases, including rheumatoid arthritis (RA) (37–40), juvenile idiopathic arthritis (41), autoimmune myasthenia gravis (42), dermatitis herpetiformis (DH) (43), psoriasis (44), systemic lupus erythematosus (SLE) (45), epidermolysis bullosa acquisita (EBA) (46), celiac disease (CD) (47), atopic dermatitis (48) and other (auto)inflammatory diseases, their pathological role is not fully understood (**Table 1**). The mechanism of anti-Hsp antibody formation also remains to be clarified. In fact, antibodies to self-Hsps are present in the sera of healthy individuals (49, 50). The presence of (auto)antibodies against Hsps in healthy people may result from at least three, mutually nonexclusive causes. First, they may represent NAbs, second, they may result from prior bacterial infection, and finally, anti-Hsp autoantibodies may arise during tissue degradation. Although the occurrence and physiological role of NAbs against autologous Hsps require further investigation (51–56), the high degree of homology between bacterial and mammalian Hsps may lead to cross-reactivity. In other words, antibodies raised against highly immunogenic bacterial Hsps during infection can recognize human Hsps homologs (the “molecular mimicry” theory) and thus become autoreactive (38). The latter mechanism may partially support the theory of an infectious basis of autoimmunity.

**Table 1:** Autoantibodies against Hsps in selected autoimmune diseases.

Antibodies to self-Hsps in autoimmune diseases	Findings	Reference
Rheumatoid arthritis		
Anti-Hdj1 (Hsp40) IgG	Unchanged levels in circulation. Association with disease activity.	(40)
Anti-Hdj2 (Hsp40) IgG	Higher levels in circulation and association with disease severity and serum levels of IL-6.	(39, 40)
Anti-Hdj3 (Hsp40) IgG	Higher levels in circulation.	(40)
Anti-Hsp60/70/90 IgG/M/A	Higher levels in circulation. Positive correlation between serum levels of anti-Hsp60 IgG and IL-4. Inverse correlation between serum levels of anti- Hsp70 IgM and TNF-α. Positive correlation between serum levels of anti-Hsp90 IgG and IFN-γ.	(37)
Juvenile idiopathic arthritis		
Anti-Hsp70 1gG/M	Higher levels in circulation. Positive correlation between serum levels of anti-Hsp70 IgG/M and disease severity.	(41)
Autoimmune myasthenia gravis		
Anti-Hsp60 IgG/M/A	Higher levels in circulation.	(42)
Dermatitis herpetiformis		
Anti-Hsp60/70/90 IgG	Higher levels in circulation. Positive correlations between serum levels of anti-Hsp60/70/90 IgG and autoantibodies against epidermal and tissue transglutaminase.	(43)
Psoriasis		
Anti-Hsp90α IgG	Higher levels in circulation. Positive correlation between serum levels of anti-Hsp90α IgG and disease severity.	(44)
Anti-Hsp70 IgG	Passive transfer of anti-Hsp70 IgG led to attenuation of disease activity and inhibition of the proinflammatory Th17 population in a psoriasis mouse model.	(22)
Systemic lupus erythematosus		
Anti-Hsp70/90 IgG	Higher levels in circulation.	(45)
Epidermolysis bullosa acquisita		
Anti-Hsp70 IgG	Higher levels in circulation. Positive correlation between serum levels of anti-Hsp70 IgG and IFN-yin EBA patients. Anti-Hsp70 IgG-treated EBA mice had a more intense clinical and histological disease activity, as well as upregulated NF-kB activation in the skin.	(46)
Celiac disease		
Anti-Hsp40/60/90 IgG	Higher levels in circulation. Positive correlation between serum levels of anti-Hsp40/60/90 IgG and autoantibodies against tissue transglutaminase.	(47)
Atopic dermatitis		
Anti-Hsp90 IgE	Higher levels in circulation.	(48)

Elevated levels of autoantibodies may regulate the inflammatory response positively or negatively, depending on the disease, isotype, and class of Hsp.

The group of autoimmune bullous skin diseases (AIBDs) is an excellent example of the involvement of autoantibodies in the development of pathology. AIBDs are characterized by the presence of well-characterized circulating and tissue-bound pathogenic autoantibodies directed against multiple skin structural proteins present in desmosomes (e.g., pemphigus vulgaris, PV), hemidesmosomes (e.g., bullous pemphigoid, BP, and epidermolysis bullosa acquisita, EBA) or against epidermal/tissue transglutaminase (eTG/tTG) present in dermatitis herpetiformis (DH), the cutaneous manifestation of celiac disease (CD) (27, 28). Circulating autoantibodies against Hsp60, Hsp70, and Hsp90 were found to be significantly increased in patients with DH (but not in patients with PV or BP) during the active phase of the disease. Patients with DH in remission were characterized by a significant reduction in the level of anti-Hsps autoantibodies, as well as autoantibodies directed against eTG/tTG (43). Similarly, the titer of circulating autoantibodies against Hsp40, Hsp60, and Hsp90 increased in patients with CD and positively correlated with circulating anti-tTG autoantibodies (47). These serological studies may suggest that anti-Hsp autoantibodies participate in the development or maintenance of DH or CD, but these observations need to be confirmed using functional assays yet.

More comprehensive studies have been performed in RA. It was found, for example, that in patients with RA, a higher level of pro-inflammatory IL-6 positively correlates with a higher titer of IgG autoantibodies directed against Hsp40. The latter correlated with disease activity or progression (39). Similarly, circulating IgG, IgM, and IgA autoantibodies directed against Hsp60, Hsp70, and Hsp90 were significantly increased in RA patients, however; statistical analysis did not show significant relationships between these autoantibodies and disease activity/progression. On the other hand, positive correlations were found between serum levels of anti-Hsp60 IgG and IL-4 or between serum levels of anti-Hsp90 IgG and IFN-γ in RA. Moreover, a significant inverse correlation was found between serum levels of anti-Hsp70 IgM autoantibodies and pro-inflammatory TNF-α in RA. These results may suggest a distinct anti-Hsps humoral autoimmune response in RA patients, which does not appear to be directly related to the pathophysiology of RA but may have a potential immunomodulatory effect in this disease (37). The importance of circulating anti-Hsp60 IgG antibodies in RA has been extensively studied in preclinical studies. Naturally occurring or acquired anti-Hsp60 (auto)antibodies directed against M. tuberculosis have been found to protect animals against adjuvant (AA)-induced arthritis. Moreover, pretreatment of rats with soluble bacterial Hsp65 protected against AA induction and was associated with suppression of IL-17 and induction of circulating anti-hsp65 antibodies. Finally, humanized anti-Hsp60 mAbs were found to be effective in protecting and inhibiting models of AA, collagen-induced arthritis, and colitis (57, 58).

It is worth mentioning an interesting comparison of the dual role of anti-Hsp70 antibodies in the development of two non-infectious inflammatory skin diseases, such as psoriasis and EBA, the latter characterized by the presence of pathogenic anti-collagen type VII (COL7) autoantibodies. The first study assessed the role of immunizing animals with Hsp70 protein on the development of psoriasis induced by topically applied IMQ. Immunization of mice with autologous or plant Hsp70 resulted in a reduction in disease activity, which was associated with the induction of Treg lymphocytes. Functional assay revealed that induced circulating anti-Hsp70 IgG in immunized animals could also inhibit disease progression, as passive transfer of anti-Hsp70 IgG led to attenuation of disease activity and inhibition of the proinflammatory Th17 population. In the same research team, it was shown that the role of the eHsp70 and anti-Hsp70 IgG antibodies is exactly the opposite. Firstly, patients with EBA were characterized by significantly increased levels of circulating anti-Hsp70 IgG autoantibodies, but this did not apply to other isotypes. The pathophysiological significance of Hsp70 and anti-Hsp70 IgG autoantibodies was demonstrated in a mouse model of EBA induced by the passive transfer of anti-COL7 IgG antibodies. Animals treated with Hsp70, or anti-Hsp70 IgG showed more intense clinical and histological disease activity. Mechanistically, anti-Hsp70 IgG enhanced neutrophil infiltration into the skin and activation of the NF-κB signaling pathway (22, 46, 59).

## 5 Perspective

**Figure 1:**
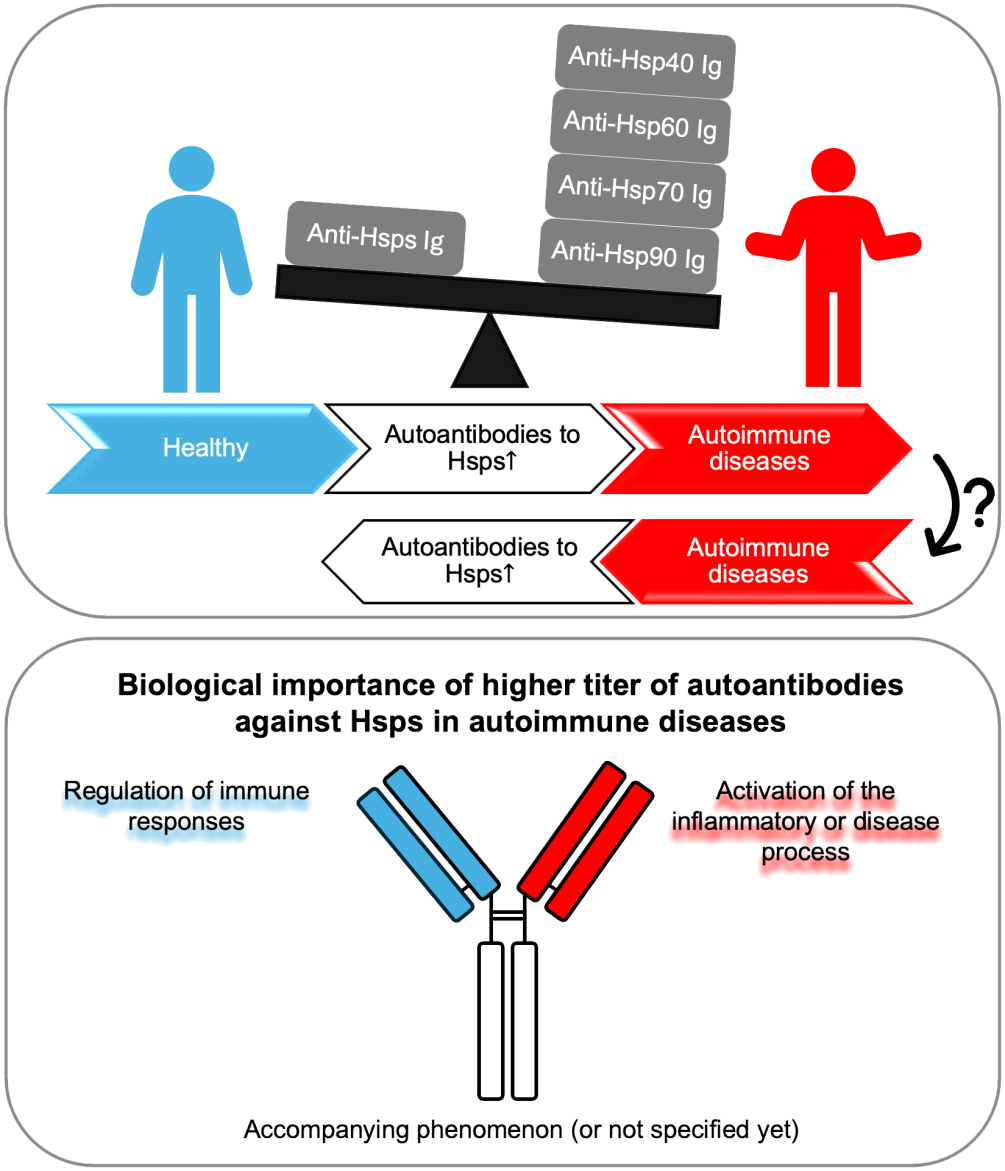
Autoantibodies against Hsp display a dual role. Autoantibodies against Hsp (produced by an unknown mechanism) may lead to inflammation and the development of autoimmune diseases, or conversely, chronic inflammation resulting from autoimmunity may lead to the production of circulating antibodies against self-Hsp. Higher titers of autoantibodies against Hsp may play a dual role - protective or pathogenic - in the context of the development of selected autoimmune diseases. Finally, in many cases, the presence of increased levels of anti-Hsp autoantibodies may be the result of general inflammation and be an accompanying phenomenon without biological significance, or their role has not been fully determined yet. Ig, immunoglobulin.

To sum up, the increased level of autoantibodies directed against Hsps may indicate their importance in the development of autoimmune diseases. In studies using patient sera, elevated anti-Hsp levels have often been associated with disease activity or progression. On the other hand, numerous functional studies conducted using cell cultures and preclinical models have shown that the biological role of anti-Hsps autoantibodies is not entirely clear. They appear to participate in some diseases as regulators of the immune response, and in others as factors promoting the inflammatory or disease process. Finally, some studies only show an increase in anti-Hsp autoantibody titers, without any association with disease activity or progression or other specific biomarkers (**Figure 1**). Therefore, based on the available literature, the importance of anti-Hsp autoantibodies in the development of autoimmune diseases cannot be clearly indicated. These differences resulted from several reasons. First, the differences concerned the type of disease and the Hsp family. It is worth emphasizing that studies suggesting the involvement of anti-Hsps in autoimmune/inflammatory diseases, based solely on observations of changes in their levels in the blood, may not be sufficient to draw final conclusions. Therefore, when planning future studies to determine the role of anti-Hsp autoantibodies in the course and development of AD, several issues should be considered. This should include selection of an appropriate animal model to confirm the significance of elevated anti-Hsp levels in patients, determination of antibody isotype, IgG subclass, Fc glycosylation/sialylation pattern, and determination of the specificity of autoantibodies directed against the autologous heat shock protein. Finally, it should be mentioned that the presence of higher titers of autoantibodies directed against various Hsps is observed in people exposed to various physical factors (e.g. high temperature or noise), chemical factors (e.g. carbon monoxide) and biological/infectious factors (e.g. malaria infection). Yet, their role in the cell/organism’s response to these stressors is unclear. In turn, it is also worth emphasizing that the lack of increase in the titer of anti-Hsp60, -Hsp70 and -Hsp90 autoantibodies in the serum of people who have had Covid-19 compared to naive people indicates that not every infectious agent causing a strong humoral immune reaction leads to production of autoantibodies against Hsp (60, 61). At this point, caution should be exercised in interpreting the results regarding the involvement of antibodies directed against self-Hsp in autoimmune diseases, as their increased level may not be related to the latter group of diseases.

## Author contributions

ST: Writing – review & editing, Writing – original draft.
